# Prevalence of taurodontism in the North Indian population

**DOI:** 10.4317/jced.51118

**Published:** 2013-10-01

**Authors:** Santosh Patil, Bharati Doni, Sumita Kaswan, Farzan Rahman

**Affiliations:** 1 Dept of Oral medicine and radiology, Jodhpur Dental College, Jodhpur National University, Jodhpur (Raj), India; 2 Dept of Oral medicine and radiology, NIMS, Jaipur (Raj), India; 3 Dept of Conservative Dentistry and Endodontics, Jodhpur Dental College, Jodhpur National University, Jodhpur (Raj), India; 4 Dept of Oral Pathology and Microbiology, Jaipur Dental College and Hospital, Jaipur (Raj), India

## Abstract

Objectives: Taurodontism affects primarily molars and premolars in both the deciduous and permanent dentition. The aim of the study was to assess the prevalence of taurodontism in the North Indian population. Study Design: 4143 patients were studied by analyzing the panoramic radiographs for the presence of taurodontism which is defined as the presence of an apically displaced pulp chamber and the tooth lacks the usual constriction at the cementoenamel junction. The age of the patients ranged from 13 to 38 years with a mean age of 21.8 years. Results: Taurodontism was found in 17 patients with a prevalence of 0.4% of which 0.21% were males and 0.19% females. Taurodonts were significantly more common in the maxilla (65.6%) than in the mandible (34.4%) (p<0.05) and the maxillary second molar (34.4%) was the most commonly involved tooth. According to the morphology hypotaurodonts were most common (75%) but there was no significant difference in males and females (p>0.05). Conclusion: Taurodontism is relatively uncommon in the North Indian population. Further large scale studies need to be carried out to assess its prevalence in the general population. A family history of other anomalies should also be considered for affected patients.

** Key words:**Taurodontism, prevalence, maxillary second molar.

## Introduction

Taurodontism is defined as a morphologic variation of a tooth that lacks the usual constriction at the cemento-enamel junction, with an apically positioned floor of the pulp chamber and furcation area resulting in shortened roots and root canals (1). Sir Arthur Keith (2) first coined the term taurodontism and defined it as “a tendency for the body of the tooth to enlarge at the expense of the roots.” It is derived from the Latin word “tauros”, meaning “bull” and the Greek word “odus” meaning “tooth”. It is similar to a tendency to assume the condition seen in the ox (3). The first case in humans was described by Pickerill (4) in 1909 and he used the term “radicular dentinoma” to describe the condition. The involved teeth appear rectangular in shape instead of tapering towards the roots. The pulp chamber is extremely large with a greater apico-occlusal height than normal, with exceedingly short roots (5).

The aetiology of taurodontism is unclear. It is thought to be caused by the failure of Hertwig´s epithelial sheath diaphragm to invaginate at the proper horizontal level, resulting in a tooth with short roots, elongated body and enlarged pulp chamber (2). Taurodontism is classified based on the relative amount of apical displacement of the pulp chamber floor as hypotaurodont, mesotaurodont and hypertaurodont. Hypotaurodont shows mild enlargement of the pulp chamber at the expense of the roots. In mesotaurodont the pulp shows moderate enlargement with short roots which are still separate, while in hypertaurodont the pulp chamber reaches the apical 3rd and then may break into two or four channels. They assume prismatic or cylindrical forms (6). Taurodontism has been reported in association with few genetic defects like hypodontia, cleft lip/palate, and certain syndromes like Down´s syndrome, Van der Woude´´ syndrome and Mohr syndrome (7-9). The literature shows wide variation on the prevalence of taurodontism. In the few studies (10,11) the prevalence has been reported to range between 5.6% and 60% of the population. The differences may also be due to ethnic variations (12). The aim of the present study was to assess the prevalence of taurodontism in the North Indian population by radiographic analysis.

## Material and Methods

The records of 4143 patients attending the Department of Oral Medicine and Radiology, Jodhpur Dental College General Hospital between September 2008 to December 2012 were investigated using panoramic radiographs for the study. Ethical clearance was obtained from the Institutional Ethics Committee. The age of the patients ranged from 13 to 38 years with a mean age of 21.8 years. All panoramic radiographs were taken with the Dentsply Gendex Orthoralix 9200 (Dentsply Asia, Milford, US), and the magnification factor was 1.23. All reported measurements were adjusted according to this factor. One group of researchers examined the radiographs at the same time on standard light boxes to determine the number and type of taurodont in the posterior teeth.

Radiographs of poor quality (distorted, elongated, over/underexposed radiographs) were excluded. Carious or restored teeth, fractured teeth, impacted teeth, teeth with fused roots and undetectable furcation areas, were not included in the study. A tooth was considered as a taurodont when there was presence of an apically displaced pulp chamber and furcation area without the usual constriction at the cementoenamel junction. All patients with taurodontism were subsequently reevaluated for any possible association with other developmental anomalies or co-existing genetic disease or syndrome. The observations were analyzed using the computer program, SPSS 12 (SPSS Inc. Chicago, USA). Chi-square test was also used to compare the prevalence of taurodontism between male and female subjects.

## Results

The study comprised of 2145 males (51.9%) and 1998 females (48.1%) with an age range of 13 to 38 years with a mean age of 21.8 years. Taurodontism was found in a total of 17 patients with a prevalence of 0.4% of which 9 (0.21%) were males and 8 (0.19%) females. There was no statistically significant difference (p>0.05) in the distribution between the sexes. Thirty two teeth of the 19146 premolar and molar teeth that were examined showed taurodontism. Taurodonts were significantly (p<0.05) more common in the maxilla (65.6%) than in the mandible (34.4%) ( Table 1). The maxillary second molar (34.4%) was the most commonly involved tooth. Only 4 premolars were involved (12.4%). According to the morphology hypotaurodonts were found in 24 teeth (75%) but there was no significant difference in males and females (p>0.05) ( Table 2). Mesotaurodonts were found in 6 teeth and 2 teeth were hypertaurodont in morphology. On reviewing the patient´s records, 2 patients showed evidence of Down´s syndrome and 1 patient was with a history of cleft lip and palate.

**Table 1:**
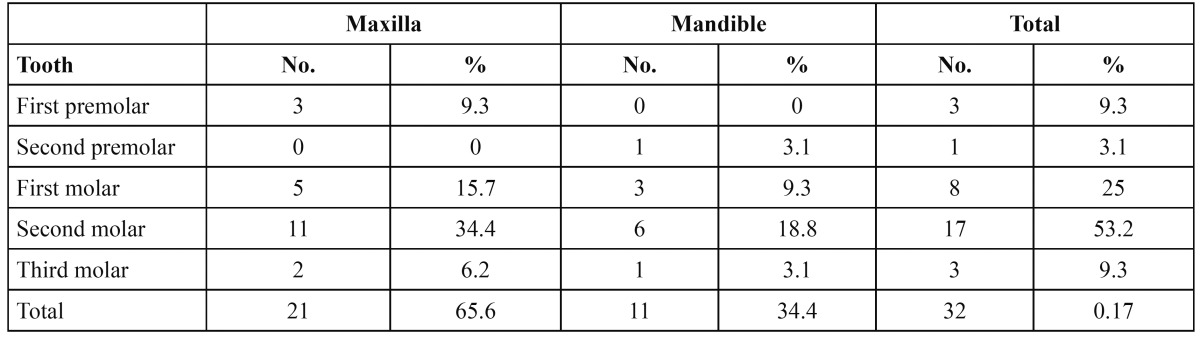
Distribution of taurodonts in the maxilla and mandible according to the affected tooth.

**Table 2:**
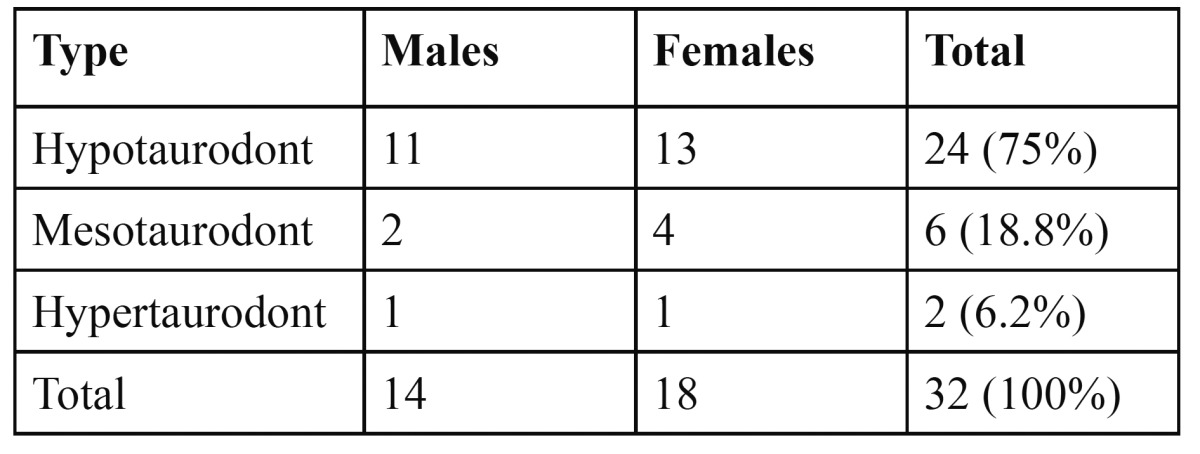
Distribution of taurodonts according to morphology by gender.

## Discussion

Taurodontism has been reported to mainly affect the posterior teeth in both the primary and permanent dentition. Although generally seen as an isolated trait, there are occasional reports of the association of taurodontism with various syndromes and developmental disorders such as hypodontia, amelogenesis and dentinogenesis imperfecta, ectodermal dysplasia, tricho-dento-osseous syndrome, Mohr syndrome, Klinefelter´s syndrome and Down´s syndrome. It is also said to be more prevalent in patients with cleft lip and palate than in normal subjects (8,9,11-14).

Taurodontism is an important dental finding which requires special attention during cavity preparation, root canal therapy and tooth extraction. Taurodontism may complicate endodontic, orthodontic and/or prosthetic treatment planning. It presents a challenge during negotiation, instrumentation and obturation during the root canal therapy for the endodontist. The extraction of a taurodont tooth may be difficult and complicated since the furcation is shifted to the apical area, especially if the roots are divergent at the apical third (15). A taurodont tooth offers favourable prognosis from periodontal point of view because these teeth have to demonstrate significant periodontal destruction before the furcation involvement. In case of prosthetic treatment of such tooth, the placement of post for tooth reconstruction may be avoided. When used as an abutment, this tooth may not offer much stability and strength, as the surface area is smaller inside the alveolus (16). Taurodontism can be interpreted from a radiograph, but some researchers have tried to develop an objective method for assessment of taurodontism (10). Although it has been shown that certain teeth that were defined as taurodont did not meet this criteria, while certain non-taurodont teeth did meet the defined criteria (17).

The literature review reveals a wide discrepancy in different populations with regards to prevalence of taurodontism. The results of the study on a group of Jordanian dental patients has shown a prevalence of 8% for individuals and 4.4% for posterior teeth (14). Ruprecht et al. (17) found an individual prevalence of 11.3% and 43.2% for molars in Saudi dental patients. Shifman and Channanel (10) reported a prevalence of 5.6% for individuals and 1.5% for posterior teeth in Israeli dental patients. Tulensalo et al. (18) reported a much higher prevalence of 60% in Finnish population, compared with 46.4% and 21.7% in a study carried out by MacDonald-Jankowski et al. (12) in young adult Chinese population. The results of the present study showed a much lower prevalence of 0.4% for individuals and the tooth prevalence as estimated to be 0.17%. These wide variations in prevalence between different populations may be attributed to ethnic variations, differences in the criteria used for interpretation of taurodontism and also the specific teeth examined.

The prevalence of taurodontism in our sample was equally distributed between males (0.21%) and females (0.19%), which is similar to the results of other studies (10,11,17). There was no significant gender difference (p>0.05). However, a higher prevalence of taurodontism was found in females in the study done by Bronoosh et al. (19) and McDonald-Jankowski et al (12). This might be attributed to an overall higher number of females in these studies. Some believe that premolars may not be affected by taurodontism and hence did not include them in their study (17,20) while others (14,21) have included premolars, similar to the present study. This is probably because premolars, except for first maxillary premolar, usually do not show more apically positioned furcation as seen in taurodontism. The current study had a higher prevalence of taurodonts in the maxillary posterior teeth, especially the second molar teeth, which is in line with the results of Laatikainen and Ranta (9), MacDonald-Jankowski et al. (12) and Tulensalo et al. (18). However, Shifman and Channanel (10) found the mandibular second molar teeth to be most frequently affected.

Hypotaurodont were the most common morphological type seen in the present study. However, no significant differences were found in the type of taurodontism between males and females (p>0.05). This was similar to the findings of Bronoosh et al. (19). Except for the classification used in a study by Keene (22), and the study of Bronoosh et al. (19) there has been no other study that compared the morphologic types of taurodontism.

Taurodontism appears most frequently as an isolated anomaly. However, it has been reported to occur with several syndromes and abnormalities (15). Many of these disorders have oral findings which can be detected on dental panoramic radiographs. Therefore, dentists may be the first to detect them. In the present study also 2 patients showed evidence of Down´s syndrome and 1 patient had presented with a history of cleft lip and palate. The simultaneous occurrence of anomalies may suggest a genetic predisposition (23). Hence, taurodontism may also provide a valuable clue for detecting any associated syndromes and other conditions.

The present study showed that the prevalence of taurodontism is much lower in the North Indian population when compared with other different populations. This shows a bias in the prevalence due to the racial differences. Regardless of the low prevalence, it is very important for a general dental clinician to be familiar with taurodontism not only due to its clinical implications but also due to probable association with related syndromes and its management.
